# Undernutrition among HIV positive women in Humera hospital, Tigray, Ethiopia, 2013: antiretroviral therapy alone is not enough, cross sectional study

**DOI:** 10.1186/1471-2458-13-943

**Published:** 2013-10-09

**Authors:** Tsegazeab Hailu Hadgu, Walelegn Worku, Desalegn Tetemke, Hailemariam Berhe

**Affiliations:** 1 Tigray Regional Health Bureau, Mekelle, Ethiopia; 2 Institute of Public Health, College of Medicine and Health Sciences, University of Gondar, Gondar, Ethiopia; 3 Department of Public Health, College of Health Sciences, Axum University, Axum, Ethiopia; 4 Department of Nursing, College of Health Sciences, Mekelle University, Mekelle, Ethiopia

**Keywords:** Undernutrition, HIV/AIDS, Food security, Dietary diversity, Antiretroviral therapy

## Abstract

**Background:**

In Ethiopia, undernutrition among women on antiretroviral therapy has been a major challenge to achieve the full impact of intervention. Twenty seven percent and 17% of reproductive age Ethiopian women are chronically malnourished and anemic, respectively. Most studies to examine risk factors have been limited to the general population and ART-naive HIV-positive women, making it difficult to generalize findings to ART-treated HIV-positive women. The objectives of this study were thus to assess nutritional status and associated factors among adult women (≥20 years) on antiretroviral therapy.

**Methods:**

From August to September we conducted an Institution based cross-sectional survey among 276 women on antiretroviral therapy in Humera Hospital, Tigray, Ethiopia. Data was collected using structured and standard face to face interview, anthropometric measurements, BD FACS (CD4 count machine) and Sysmex-21 (hemoglobin analyzer). Logistic regression was done using SPSS version 16 to identify factors that are associated with nutritional status.

**Results:**

The prevalence of under nutrition (Body mass index < 18.5 kg/m^2^) Was 42.3% (95% CI: 37.4% - 47.3%). Severe, moderate and mild under nutrition was detected on 12%, 10% and 20.3% respondents, respectively. The prevalence of wasting (percentage body weight loss >5%) was 75% (95% CI: 70.4% - 79.2%). Severe wasting was accounted for 26.9% of respondents.

In the multivariate analysis, Household food insecurity [AOR = 1.85; 95%CI 1.16, 2.86], inadequate dietary diversity [AOR = 1.19; 95%CI 1.08, 1.75], anemia [AOR = 1.67; 95%CI 1.05, 2.65] and absence of nutritional support [AOR = 0.34 95%CI 0.22, 0.54) were found to be independent predictors of under-nutrition.

**Conclusion:**

HIV/AIDS is associated with an increased burden of undernutrition even among ART treated women in Humera Hospital, Tigray, Ethiopia. In addition to ART among HIV positive women interventions to ameliorate poor nutritional status may be necessary in this and similar settings. Such interventions aimed at improving household food security, dietary diversity, micronutrient supplementation, proper use of therapeutic food, as well as treating oral candidiasis.

## Background

The HIV epidemic remains one of the main public health challenges especially in low and middle income countries. At the end of 2010, an estimated 34 million people were living with HIV globally. There were 2.7 million new HIV infections in 2010, including 390 000 among children less than 15 years. The annual number of people dying from AIDS related causes worldwide was 1.8 million in 2010 [1].

In sub-Saharan African countries, where most of the people newly infected with HIV live, an estimated 1.9 million became infected in 2010. Globally, women constituted half (50% [48–53%]) of the adults (15 years and older) living with HIV in 2010, according to UNAIDS estimates. In sub-Saharan Africa, 1.4 times more adult women than men were living with HIV in 2010. Women comprised 59% [56–63%] of adults living with HIV in sub-Saharan Africa in 2010 as they have for most of the past decade [1,2].

In Ethiopia, the prevalence of HIV in adults age 15-49 is 1.5 percent. HIV prevalence among women 15-49 ages is 1.9 percent. While for men 15-49, it is 1 percent. HIV prevalence levels in Ethiopia rise with age peaking among women in their late 30s and among men in their early 40s [3]. Antiretroviral therapy coverage in Ethiopia was 50-69% with an estimated 222 723 people receiving ART in December 2010 [1]. The availability of HAART has extended the lives of many people with HIV/AIDS and greatly reduced morbidity and deaths due to AIDS and related complications. However, HAART medications can cause problems that will create troubling symptoms like appetite loss, nausea and vomiting, diarrhea, bone death and destruction, lipodystrophy, liver toxicity, pancreatitis, and insulin resistance and diabetics [4].

About 47% of the Ethiopian population is estimated to live below the poverty line. Malnutrition is one of the main health problems facing children and women in Ethiopia. The country has the second highest rate of malnutrition in Sub-Saharan Africa. Ethiopia faces the four major forms of malnutrition: Acute and chronic malnutrition, Iron deficiency Anemia, Vitamin A deficiency, and Iodine deficiency Disorders [5]. The Ethiopia demographic health survey 2011 has shown 27% of reproductive age women are chronically malnourished (BMI < 18.5), 6% are overweight or obese and 17% anemic (BMI >25 kg/m^2^) [3].

Malnutrition is the outcome of imbalance of nutrient intake with physiological demand for growth, maintenance and reproduction. People living with HIV are at a higher risk of malnutrition as HIV infection reduces food intake, lowering food absorption and increases nutritional needs even during early stages of HIV infection, when no symptoms are apparent. The demand increases significantly during the course of the infection posing additional challenges to people living with HIV and their care providers. Food and nutritional intake can affect adherence to antiretroviral drugs (ARVs) as well as their effectiveness. Food insecurity and inadequate knowledge of good nutrition thus, impede management of the disease, particularly in resource-constrained settings where HIV is prevalent and health care services remain inadequate [6]. Food insecurity is both an outcome of and a contributor to the HIV/AIDS pandemic. Families with a member living with HIV are more apt to be poor and food insecure. Being infected with HIV limits productivity, leading, in turn, to loss of income while increasing health care costs. People living with HIV/AIDS often identify their highest priority need as food, and HIV affects food security [7].

Malnutrition among women remains a major challenge to achieve the full impact of interventions aimed at improving their quality of life, productivity and survival. Nutrition is an important component of comprehensive care for HIV-infected women and is particularly so in resource-limited settings where malnutrition and food insecurity are endemic. Preexisting malnutrition compromises the immune system. The cellular effects of malnutrition on the immune system are similar to those of HIV [8]. The relationship between HIV/AIDS and malnutrition presents a classic example of the well recognized “viscous cycle” of immune dysfunction, infectious disease, and malnutrition [9]. Nutritional issues are common in HIV disease. At some point, almost everyone living with HIV will face challenges in maintaining good nutrition. Problems are related to HIV infection itself, food security and to the effects of anti-HIV therapy [10]. Certain ARVs affects nutrient utilization by affecting nutrient absorption, metabolism, distribution, or excretion. For example, certain protease inhibitors (PI), such as ritonavir and nelfinavir, can cause changes in the metabolism of lipids (fats), resulting in an elevation in blood cholesterol and triglyceride levels [11].

Malnutrition contributes to immune system impairment, making the body vulnerable to frequent illness and increasing its energy and nutrient demand, thereby accelerating disease progression. All adult people living with HIV require 10% more energy when asymptomatic and 20–30% more when symptomatic. Children who are experiencing weight loss need between 50% and 100% more energy every day. There is no evidence that people living with HIV/AIDS require more protein and micronutrient than uninfected people. Any micronutrient supplementation should not be more than one RDA unless the client is deficient [11,12] Additional file 1[13]. Therefore, the study aimed to determine nutritional status and identify factors associated with malnutrition among women on ART at Humera Hospital, Western Tigray, Ethiopia.

## Methods

### Study design, area and study population

This institution based cross-sectional study was conducted on 376 women on ART from August 11 to September 2012 in Humera Hospital ART clinic. It is located in north Ethiopia about 950 km from Addis Ababa. Humera town is growing and has 24,195 populations and with 2.9% HIV prevalence which is higher than the national average. It has one Hospital serving the population of the town and outside.

ART service at Humera hospital had started in 2005 by the government in collaboration of partners and has two clinics: adult, and PMTCT clinic. This Hospital serves people from the zone as well as other neighboring country, north Sudan. In this zone there are two district hospitals, Dansha Hospital and Humera Hospital. In Humera Hospital adult ART clinic, there were about 1455 clients on ART care among whom 850 patients women. This study was included all women on ART excluded pregnant and lactating women for unclear anthropometric cut of values. The Study population consisted of adult (>20 years) women clients following ART in the catchment area.

### Sample size calculation

The sample size of the study was calculated using the formula for estimation of single population proportion by the assumption of: p = 37% proportion of Ethiopian women with Undernutrition due to HIV in a study conducted among women of sub-Saharan Africa in 2008. With an assumption of margin of error 0.05 at 95% confidence level and 5% non response rate, the sample size was 376. Sample size for selected potential variables Hg, food security and dietary diversity were calculated and below 376 (Table 1). Sample size reduction formula was not used so as to have sufficient sample.

**Table 1:** Summary of sample size calculation for associated factors with malnutrition among women living with HIV/AIDS using Open Epi, open source calculator—SSCC

**Variables as exposure**	**Assumptions**					**Sample size (controls + cases)**
	**OR**	**P**	**Ratio**	**Power**	**CI**	
**Anemia**	2	33%	1:1	80%	95%	298
**Food insecurity**	1.9	41%	1:1	80%	95%	324
**Low dietary diversity**	2	33%	1:1	80%	95%	298

OR = odds ratio, p = proportion, Ratio = cases to control & CI = confidence interval.

Study subjects were selected by systematic random sampling technique; taking every other woman until the desired sample size of 376 was reached (interval size, K = N/n = 850/376 = 2).

### Measurements

Economic status was assessed by using a Weighted Wealth Index incorporating household assets ownership, housing characteristics, land ownership, tractor, livestock and electricity. Dichotomous variables were constructed and factor analysis using principle component analysis (PCA) used to reduce 12 items to 5 (loaded as factor 1). Factor loadings were used as item weights, which were totaled to yield the wealth index for each household. The total Weighted Wealth Index score was then equally divided into 5 quintiles designating fifth (highest), fourth, third, second and low (poor economic status) [13].

Food security was assessed using complete form of HFIAS, 18 items scale developed by USAID [14,15] which was validated in Addis Ababa in 2008 among female HIV/AIDS home based care givers [14].

A total dietary diversity score was calculated from a recalled list of food items consumed over the previous day [16]. Based on a set list of 9 food items, a score lower than 4 was classified as low dietary diversity [17,18].

Percentage of body Weight Loss was calculated as: (Usual body weight-current weight)/usual body weight ×100. It was classified in to four normal (<5%), mild (5-10%), moderate (11-20%) and severe (>20%) [19].

We measured height using seka measuring rod calibrated to 0.5 cm. The weight and height were converted to BMI using SPSS version 16 according to WHO 1992 reference value [20,21]. The women’s weights were measured using standardized digital seka weight scale calibrated to 0.1 kg [22].

CD^4^ was measured with BD FACS machine (US) and categorized according to clinical significance. Hemoglobin was measured with sysmex-21 and categorized in to normal (≥12 g/dl) and anemic (<12 g/dl) based on WHO 1992 reference value for non pregnant and lactating mother.

### Data collection

Two medical doctors, two BSc nurses, one medical laboratory technologist and one data clerk were employed and trained for two days on interviewing technique and questionnaire content. They thereafter pre-tested the questionnaire under supervision of the first author. Data were collected by face-to-face interview, laboratory analysis, physical examination and relevant medical data were retrieved from medical files in August 11 and September 2012.

### Data analysis

We analyzed data from 276 ART treated HIV positive women. Descriptive analysis was conducted by Chi-square. Both bivariate and multiple logistic regression analyses were used to examine associations of various factors with underweight and wasting. Multiple logistic regression analyses were used to examine the associations of factors with underweight and wasting. In these models, we controlled for womens’ ART duration, diarrhea, tuberculosis, oral candidiasis, CD4 level, Hg level, dietary diversity, nutritional support, eating problem, and food security. Multicollinearity in both models was checked by examining the standard errors for regression coefficients [23]. Statistical significance was set at 95% CI and P-value <0.05. Analysis was conducted using SPSS Inc. version 16.

### Ethical consideration

This study was approved by the Institutional Review Boards of the University of Gondar. Permission to conduct the research was granted by the relevant health departments of Tigray regional health bureau and by the chief executive officer in the Hospital. Participation was voluntary, confidentiality ensured, and informed consent secured before the start of each interview, blood drawing and retrieving medical record.

## Results

### Socio-demographic characteristics

A total of 376 adult women respondents on ART were included with a response rate of 100%. The mean ages of respondents were 32.5 ± 8 SD years. Two hundred thirty four (62.2%) of respondents were not literate (Table 2).

**Table 2:** Socio-demographic characteristics of women on ART at Humera zonal Hospital, Tigray; August 2012, (n = 376)

**Characteristics**	**Frequency**	**Percent**
**Age**		
20-29	152	40.4
30-39	143	38.0
40-49	60	16.0
50^+^	21	5.6
**Residence**		
Urban	340	90.4
Rural	36	9.6
**Marital status**		
Married	185	49.2
Single	28	7.4
Divorced	103	27.4
Widowed	60	16.0
**Ethnic group**		
Tigrian	252	67.0
Amhara	124	33.0
**Religion**		
Orthodox	363	96.6
Muslim	13	3.4
**Education**		
Not literate	234	62.2
Read and write	41	10.9
Primary	77	20.5
Secondary and above	24	6.4
**Occupation**		
Farmer	94	25.0
Merchant	85	22.6
Daily labourer	133	35.4
Commercial sex work	16	4.3
No job	34	9.0
House servants	14	3.7
**Wealth index**		
First quintile	53	14.1
Second quintile	99	26.3
Third quintile	88	23.4
Fourth quintile	70	18.6
Fifth quintile	66	17.6

### Medical and related problems of respondents

Most clients, 141 (39.6%) were on ART regimen 1a (D4T-3TC-NVP) followed by 1c (AZT-3TC-NVP), 108 (30.3%). The mean duration of clients on ART was 3.4 ±2SD years (Table 3).

**Table 3:** Medical and other related problems of women on ART at Humera zonal Hospital, Tigray; August 2012, (n = 376)

**Disease/problem**	**Frequency**	**Percent**
**Eating problem**		
Yes	74	19.7
No	302	80.3
**Types of eating problem**		
Swallowing difficulty	38	10.1
Loss of appetite	33	8.8
**Current or past OI**		
TB	98	26.1
Candidiasis	53	14.1
Toxoplasmosis	11	2.9
Diarrhea	271	72.1
**Current condition**		
Improved	359	95.4
Same	16	4.3
Deteriorated	1	0.3
**Clinical stage**		
Stage-I	127	33.8
Stage-II	27	7.2
Stage-III	138	36.7
Stage-IV	84	22.3
**Adherence**		
High	318	84.6
Moderate	47	12.5
Low	11	2.9

**CD4 and Hgb level:** the median CD4 and mean Hgb level of respondent were 435 cells/μl and 11.5 g/dl with ±1.5 SD, respectively (Table 4).

**Table 4:** CD4 and Hgb level of women on ART at Humera Zonal Hospital, WesternTigray; August 2012, (n = 376)

**Immunohematology variables**	**Frequency**	**Percent**
**CD4 level**		
< 200 cells/μl	68	18.1
200-350 cells/μl	96	25.5
>350 cells/μl	212	56.4
**Hgb level**		
< 12 g/dl	250	66.5
≥ 12 g/dl	126	33.5

### Dietary diversity, household food security and nutritional support (plump nut) profile: (Table 5)

**Table 5:** Nutritional status of women on ART at Humera Zonal hospital, WesternTigray, August 2012, (n = 376)

**Variables**	**Frequency**	**Percent**
**Household food security**		
Secured	224	59.6
Insecure	152	40.4
**Women dietary diversity**		
Adequate	176	46.8
Inadequate	200	53.2
**Nutritional support**		
Yes	147	39.1
No	229	60.9

**Figure 1:**
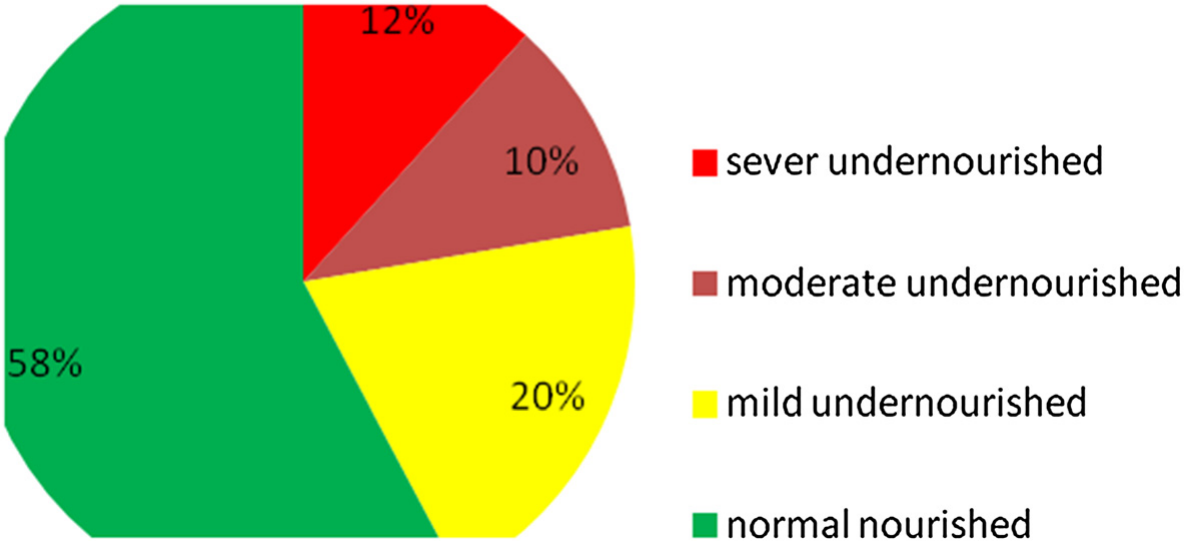
Nutritional status (BMI) of adult women on ART at Humera Hospital, Tigray, August 2012, (n = 376).

**Figure 2:**
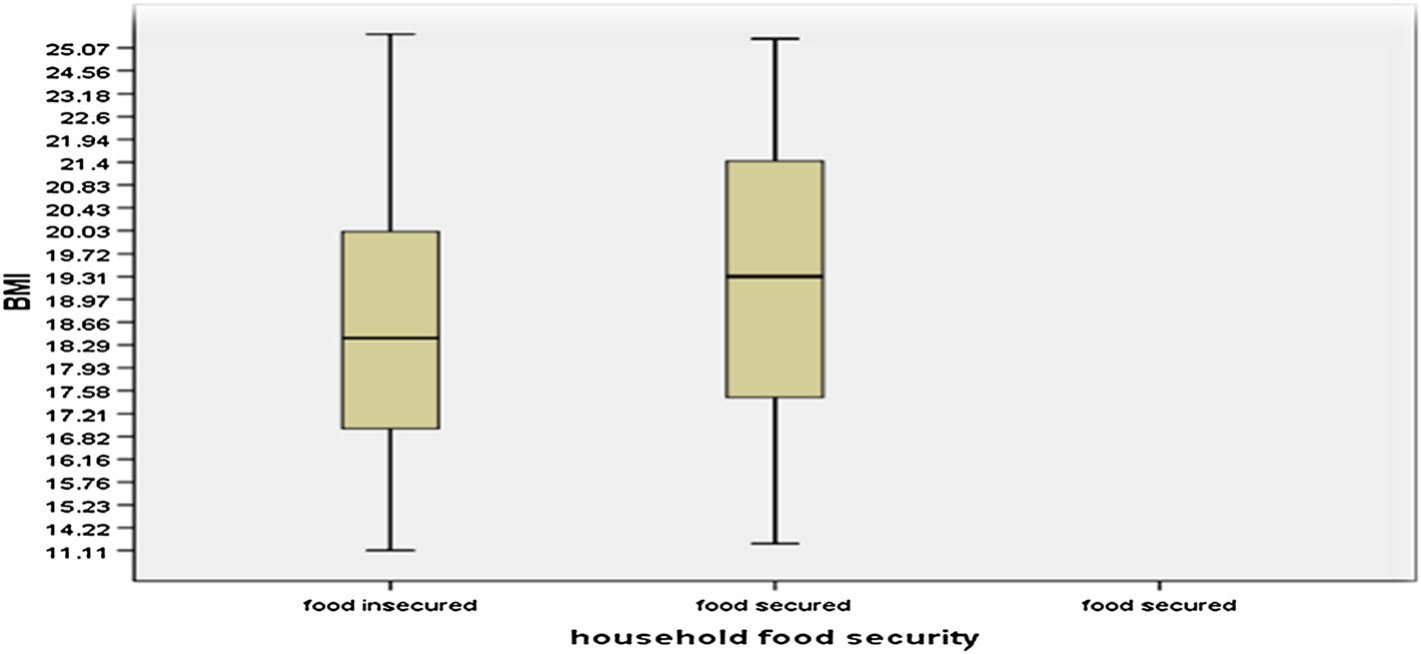
Box plot of body mass index by household food security status of adult women on ART at Humera Hospital, Tigray, August 2012, (n = 376).

**Nutritional status of women on ART**: the prevalence of overall Undernutrition (BMI <18.5 kg/m^2^) was 42.3% (95% CI: 37.4% - 47.3%). The mean BMI of the respondents was 19 kg/m^2^ with SD of ±2.5. Using percentage body weight loss, the prevalence of malnutrition was 75% (95% CI: 70.4% - 79.2%) with mild malnutrition (BWL between 5-10%) 14.6%, moderate (10-20%) 33.5%, and sever (>20%BWL), 26.9%. Ninety four (25%) of women have no malnutrition (BWL < 5%) (Figures 1 and 2).

Multivariate logistic regression analysis was done looking for association of variables with nutritional status (BMI). Nutritional status was significantly associated with household food security, nutritional support (plumpy nut), women dietary diversity and hemoglobin level (Table 6).

**Table 6:** Logistic regression of nutritional status (BMI) and predictors among women on ART, Humera Hospital, Western Tigray, August 2012, (n = 376)

**Predictors**			**Malnutrition**				**COR (95%CI)**	**AOR (95%CI)**
	**Yes**	**No**	**Total**					
ART in month								
≤ 3	14	13	27				1.19 (.54,2.61)	
3-6	7	5	12				1.55 (.48,4.97)	
≥ 6	160	177	337				1	
Chronic diarrhea								
Yes	133	138	271				1.14 (.73,1.79)	
No	48	57	105				1	
Tuberculosis								
Yes	50	48	98				1.17 (.74,1.85)	
No	131	147	278				1	
CD4 level								
< 200 cells/μl	39	29	68				1.54 (.88,2.66)	
200-350 cells/μl	43	53	96				0.93 (.57,1.50)	
> 350 cells/μl	99	113	212				1	
DDS								
Adequate (≥4)	61	115	176	120	80	200	1	1
Inadequate (<4)							2.83 (1.86,4.30)	1.19 (1.08,1.75)*
Nutritional support								
Yes	90	57	147				1	1
No	91	138	229				0.42 (.27,.67)	0.34 (.22,.54)* *
HFSS (0-9)								
Secured	98	126	224				1	1
Insecure	83	69	152				1.55 (1.02,2.34)	1.85 (1.16,2.86)* *
Hgb								
Normal (≥12 g/dl)	51	75	126				1	1
Anemic (<12 g/dl)	130	120	250				1.59 (1.03,2.46)	1.67 (1.05,2.65)*

DDS = Dietary diversity score, *p < 0.05 (2-tailed), * *p < 0.001(2-tailed), HFSS = household food security status and Hgb = hemoglobin.

N.B: Hosmer and lemeshow test was at p = 0.99.

Stepwise (Backward LR) was used in logistic regression.

On the other hand wealth index, nutritional counseling, duration of ART, regimen type, clinical stage, CD4 level, adherence, eating problem, opportunistic infections and socio-demographic variables were not associated with nutritional status.

Household food security was positively associated with nutritional status. Women who were household food insecure were 1.85 times more likely to be undernourished (BMI < 18.5 kg/m^2^) as compared to those who were household food secure (AOR = 1.85, 95% CI: 1.16, 2.86). Nutritional care and support were negatively associated with nutritional status (BMI). Participants who were not taking nutritional care and support were 0.34 times less likely to be undernourished than those who were taking nutritional care and support (AOR = 0.34, 95% CI: 0.22, 0.54) (Table 6).

There was a statistically significant positive association between nutritional status (BMI) and women's dietary diversity. Clients who were taking inadequate diversified food were 1.19 times more likely to be undernourished (BMI < 18.5 kg/m^2^) as compared to those who were taking adequate diversified food (AOR = 1.19, 95% CI: 1.08, 1.75). Hemoglobin level also positively associated with nutritional status of women. Women who were anemic were 1.67 times more likely to be undernourished (BMI < 18.5 kg/m^2^) than those with normal hemoglobin level (AOR = 1.67, 95% CI: 1.05, 2.65) (Table 6).

In Bivarate logistic regression analysis to see the relationship between nutritional status (%BWL) and covariates, there was a statistically significant association among nutritional support, eating problem, household food security and oral candidiasis.

In multivariate logistic regression household food security and oral candidiasis were statistically significantly associated with nutritional status (%BWL). Respondents with household food insecurity were 1.90 times more likely to have significant weight loss as compared to those who were household food secure (AOR = 1.90, 95% CI: 1.11, 3.25). Respondents who were infected with oral candidiasis were 2.42 times more likely to be wasted than who were free of oral candidiasis (AOR = 2.42, 95% CI: 1.03, 5.25) (Table 7).

**Table 7:** Logistic regression of nutritional status (%BWL) and predictors among women on ART at Humera Hospital, Western Tigray, August 2012, (n = 375)

**Predictors**	**Wasting (≥5%BWL)**			**COR (95%CI)**	**AOR (95%CI)**
	**Yes**	**No**	**Total**		
**Nutritional support**					
Yes	119	28	147	1.84 (1.12, 3.04)	
No	159	69	228		
**Eating problem**					
Swallowing difficulty	35	3	38	4.53 (1.36, 15.12)	
Loss of appetite	24	9	33	1.04 (.46, 2.32)	
No	219	85	304	1	
**Oral candidiasis**					
Yes	46	7	53	2.55 (1.11, 5.56)	2.42 (1.03, 5.25)*
No	232	90	322	1	1
**HFSS (0-9)**					
Secure	153	71	151	1	1
Insecure	125	26	224	2.23 (1.34, 3.71)	1.90 (1.11, 3.25)*

HHSS = household food security score, ^R^ Reference category and * Significant at p < 0.05 (2 tailed).

N.B: Hosmer and lemeshow test was at p = 0.162.

Stepwise (Backward LR) was used in logistic regression.

## Discussion

The study aimed to determine nutritional status and identify factors associated with malnutrition among women on ART at Humera Hospital, Western Tigray.

Results showed that women on antiretroviral therapy suffer from undernutrition (BMI < 18.5 kg/m^2^) and wasting (%BWL > 5%). It is not always possible to identify one single cause as the main contributor to declining nutritional status or malnutrition in HIV infected patients. The etiology of HIV-associated wasting is multifactoral, and causes may include socioeconomic status, access to care, cultural practices, psychological factors, and medical complications and therapies for HIV infection [24]. Despite tremendous advances in care for human immunodeficiency virus (HIV) infection and increased funding for treatment, morbidity and mortality due to HIV/AIDS in developing countries remains unacceptably high. A major contributing factor is that >800 million people remain chronically undernourished globally, and the HIV epidemic largely overlaps with populations already experiencing low diet quality and quantity [25].

The prevalence of overall undernutrition (BMI < 18.5 kg/m^2^) in this study was 42.3% (95% CI: 37.4% - 47.3%). In this regard earlier studies documented the magnitude of Undernutrition (BMI < 18.5 kg/m^2^) in Gondar University Hospital in 2007 (27.8%) [26], St. Peter Hospital in Addis Ababa in 2008 (25%) [27] and Addis Ababa city in 2009 (18%) [28]. The result of the present study is higher than the results of previous studies. This high rate of Undernutrition in this study could be due to high prevalence of household food insecurity (40.4%) leading to lack of access to adequate, safe and nutritious food resulting to Undernutrition. There were also a large number of subjects (53.2%) taking inadequate dietary diversified food which reflects low micronutrient intake; it may contribute to the pathogenesis of HIV through increasing oxidative stress and compromised immunity and indirectly resulting in Undernutrition. On the other hand, this discrepancy may be due to the present study used women only which can increase the prevalence of undernutrition (BMI < 18.5) since BMI is affected by sex, age and muscle mass [28]. The second possible reason for the high prevalence of Undernutrition in the present study could be due to high sample size compared to AA city (153 subjects) [29]. The third possible reason for low prevalence of Undernutrition in Addis Ababa could be, respondents in Addis Ababa may have a better household food security, dietary diversity, nutritional care and support and overall better ART follow up and monitoring resulting in alleviation of Undernutrition as Addis Ababa is civilized city with good climate and possibly better clinicians compared to present study area which is harsh environment.

A meta-analysis study conducted in 11 sub-Saharan countries reported that the pooling prevalence estimates of HIV-related Undernutrition of 10.3% which is lower than this study [30]. The difference could be due to the meta-analysis study participants were all HIV women clients which includes women not eligible for ART that can undermine the prevalence of Undernutrition since most women who are not eligible to ART are in a good nutritional and immunological status.

Moreover, a similar study conducted in 2010 in Glasgow, United Kingdom among HIV patients on HAART founded prevalence of Undernutrition 29.7% (female, 23.1% and male 6.6%). This difference between the findings on Undernutrition could be due to the high prevalence of Undernutrition among the population in general in Ethiopia compared to the population in the United Kingdom, a developed nation with a better care for HIV clients, possible earlier presentation to clinics and better baseline nutritional status [31,32].

Based on percentage BWL assessment, the prevalence of all levels of malnutrition was 75% with mild, moderate and severe malnutrition being 14.6%, 33.5%, and 26.9%, respectively. It indicated that HIV contributes to loss of body weight on top of other cause of malnutrition. This result is higher as compared to a study in Gondar University Hospital (75% vs. 60.9%) both in overall and different level of malnutrition [26].

This may be because of a better management and care of HIV in the University Hospital and due to the study subjects in the present study who were all women; women being more vulnerable to wasting than men due to women have high fat tissues and low amount of lean mass tissue compared to men. This study is demonstrated this concept by revealing high prevalence of wasting among women on ART.

Likewise a study done in Free State Province of South Africa found the prevalence of wasting, defined as a body mass index (BMI) <18.5 kg/m^2^ in adults with advanced HIV infection in sub-Saharan Africa is 20-40% which is lower than the present study. The difference between the findings is may be due to the deference in method of measuring wasting, the study in South Africa measures wasting with BMI <18.5 kg/m^2^, this may under estimate the prevalence of wasting as women with normal BMI can be wasted in relative to their usual body weight [33].

Though ART expected to reverse HIV associated anemia, in this study there were significant number of anemic (66.5%) respondents and anemia was a statistically significantly associated with Undernutrition (AOR = 1. 67, 95% CI: 1.05, 2.65). This result is higher than a study conducted among women of sub-Saharan Africa living with HIV (excluding pregnant and lactating) that is 37% [34]. This may be due to high burden of hemoparasitic infection especially malaria as the study area is endemic for malaria throughout the year, inadequate dietary diversified food, household food insecurity, intestinal parasitosis and drug side effect which all lead to anemia.

The results of this study suggested that household food insecurity and women dietary diversity were significantly associated with Undernutrition (BMI < 18.5) among women on ART independent of nutritional support, anemia, duration of ART, CD4 level, TB and other factors (AOR = 1.85, 95% CI: 1.98, 2.86 and AOR = 1.19, (95% CI: 1.08, 1.75), respectively. These results were consistent with an earlier similar study conducted in Uganda in 2012 among PLWHA on ART that those who were food insecure were more likely to be undernourished with AOR = 1.92 [24]. However, a study conducted in Rwanda among HIV positive and HIV negative women founded Undernutrition was not associated with food insufficiency or dietary diversity [25].

This contradiction could be due to the difference in assessment tool; the study conducted in Rwanda was used a single question to assess food insufficiency which is not appropriate to assess food insufficiency as food insufficiency (food insecurity) has standard multiple questions and women dietary diversity questionnaire is better than household dietary diversity to assess dietary diversity among women [19-35].

Other important finding of this study, which has implication for practical programming, is the negative relationship of nutritional support (plupy nut) and Undernutrition. A prospective cohort study in Haiti done in 2010 demonstrated that food assistance among PLWHA on ART at a base line of BMI = 20.4 kg/m^2^ significantly improved their BMI in 12 months support [36]. This disagreement between the findings, it can confidently be said that households with food insecurity was probably shared nutritional support (plupy nut) among household members or sold to get money. This finding is not surprising as many studies in Uganda and elsewhere shows that households of women living with HIV/AIDS have a higher risk of household food insecurity [35].

In the present study, wasting was statistically significant with household food security and oral candidiasis. This result agreed with a study done in Malawi in 2010 [28].

The pathophysiological mechanisms underlying with HIV wasting syndrome are related to four major factors including inadequate nutrient intake, nutrient malabsorption, disturbances in metabolism and catabolic state induced by opportunistic infections [32].

Results may not represent the situation of the whole year, for undernutrition (18.5 kg/m^2^) is affected seasonally in Ethiopia. As the calculation of BWL, assessment of household food security and dietary diversity depends on memory, there may be a possibility of recall bias.

## Conclusion

The prevalence of Undernutrition (BMI < 18.5 kg/m^2^), anemia, wasting (%BWL > 5%) and household food insecurity are very high among women on ART despite of HAART in this area. Weight loss is a major problem among women on ART.

Clients with household food insecurity, inadequate diversified diet, anemia and nutritional support (plumpy nut) are more likely to be undernourished. Participants with household food insecurity and chronic oral candidiasis are more likely to be wasted.

Attention needs to be given to improve household food security of women living with HIV/AIDS in addition to ART, OIs treatment and supplementation of RUTF. Attention needs to be given to micronutrient supplementation as well.

Consistent and proper nutritional assessment and diagnosis and treatment of anemia should be a vital part of HIV management and a prerequisite to the planning of general nutritional care and support for women on ART.

Women on ART need to be organized and create self reliance on food in order to improve their household food security and feed themselves and their household members.

The effect of food by prescription on ameliorating Undernutrition, improving treatment response and quality of life which is not included in this study needs prospective Cohort study. Randomized controlled experimental study design is needed to know the effect of nutritional support (plumpy nut) on nutritional status of women on ART independent of food security status.

## Competing interests

We declare that we do not have competing interests.

## Authors’ contributions

TH participated in the conception, design of the study and coordinated the data collection. WW analyzed the data; DT drafted the manuscript. HB involved in the conception, design of the study, field work and review of the manuscript. All authors read and approved the final manuscript.

## Pre-publication history

The pre-publication history for this paper can be accessed here:

http://www.biomedcentral.com/1471-2458/13/943/prepub

## Supplementary Material

Additional file 1 HIV clinical staging criteria. Click here for file
