# Supplementary material for: Undernutrition among HIV positive women in Humera hospital, Tigray, Ethiopia, 2013: antiretroviral therapy alone is not enough, cross sectional study
Source: BMC Public Health. 2013 Oct 9;13:943. doi: 10.1186/1471-2458-13-943 (PMC3852443; doi:10.1186/1471-2458-13-943)
Supplement: Additional file 1 — HIV clinical staging criteria. [file 1471-2458-13-943-S1.doc]

## Additional file 1: HIV clinical staging criteria

- - - 1. **WHO Staging System for HIV Infection and Disease in Adults and Adolescents**

- - 1. **Detailed description of the clinical stages of HIV/AIDS**

**Clinical stage 1:** a person with confirmed HIV infection who is asymptomatic and/or Persistent generalized lymphadenopathy (PGL)

**Clinical stage 2:** A person with confirmed HIV infection and having: Moderate unexplained weight loss (<10% of presumed or measured body weight), Recurrent respiratory tract infections (RTIs, sinusitis, bronchitis, otitis media, pharyngitis), Herpes zoster, Angular cheilitis, recurrent oral ulcerations, Papular pruritic eruptions, Seborrhoeic dermatitis and Fungal nail infections of fingers

**Clinical stage 3:** Conditions where a presumptive diagnosis can be made on the basis of clinical signs or simple investigations: Severe weight loss (>10% of presumed or measured body weight), Unexplained chronic diarrhea for longer than one month, Unexplained persistent fever (intermittent or constant for longer than one month), Oral candidiasis, Oral hairy leukoplakia, Pulmonary tuberculosis (TB) diagnosed in last two years, Severe presumed bacterial infections (e.g. Pneumonia, empyema, pyomyositis, bone or joint infection, meningitis, bacteraemia), Acute necrotizing ulcerative stomatitis and gingivitis or periodontitis

**Clinical stage 4:** Conditions where a presumptive diagnosis can be made on the basis of clinical signs or simple investigations*:* HIV wasting syndrome, Pneumocystis Carinii pneumonia, Recurrent severe or radiological bacterial pneumonia, Chronic herpes simplex infection (orolabial, genital or anorectal of   more than one month’s duration), Oesophageal candidiasis, extra pulmonary TB, Kaposi’s sarcoma, Central nervous system (CNS), toxoplasmosis, and HIV encephalopathy

**Conditions where confirmatory diagnostic testing is necessary:** Extra pulmonary cryptococcosis including meningitis**,** Disseminated non-tuberculous mycobacteria infection**,** Progressive Multifocal Leukoencephalopathy (PML)**,** Candida of trachea, bronchi or lungs, Cryptosporidiosis, Isosporiasis, Visceral herpes simplex infection, Cytomegalovirus (CMV) infection (retinitis or of an organ other than liver, spleen or lymph nodes), Any disseminated mycosis (e.g. histoplasmosis, coccidiomycosis, penicilliosis), Recurrent non-typhoidal salmonella septicaemia, Lymphoma (cerebral or B cell non-Hodgkin), Invasive cervical carcinoma, Visceral leishmaniasis, CD4 and Hgb.
